# DNA methylation mediates the multiple sclerosis onset risk associated with HHV-6 DNA positivity

**DOI:** 10.1093/eep/dvag016

**Published:** 2026-05-25

**Authors:** Alex Eisner, Steve Simpson-Yap, Daniel J Park, Ellen Morwitch, Samuel Tanner, Vicki E Maltby, Ingrid van der Mei, Jeannette Lechner-Scott, Rodney J Scott, Simon A Broadley, Rod A Lea, Pernilla Stridh, Tomas Olsson, Maja Jagodic, Lars Alfredsson, Bruce V Taylor, Ausimmune Investigators Group, Caron Chapman, Alan Coulthard, Keith Dear, Terry Dwyer, Trevor Kilpatrick, Robyn Lucas, Tony McMichael, Anne-Louise Ponsonby, Bruce Taylor, Patricia Valery, Ingrid van der Mei, David Williams, Anne-Louise Ponsonby

**Affiliations:** The Florey Institute of Neuroscience and Mental Health, The University of Melbourne, Parkville, 3010, Australia; The Florey Institute of Neuroscience and Mental Health, The University of Melbourne, Parkville, 3010, Australia; Neuroepidemiology Unit, Melbourne School of Population & Global Health, The University of Melbourne, Carlton, 3053, Australia; MS Research Flagship, Menzies Institute for Medical Research, University of Tasmania, Hobart, 7000, Australia; Melbourne Bioinformatics, The University of Melbourne, Parkville, 3051, Australia; Department of Biochemistry and Pharmacology, The University of Melbourne, Parkville, 3051, Australia; The Florey Institute of Neuroscience and Mental Health, The University of Melbourne, Parkville, 3010, Australia; The Florey Institute of Neuroscience and Mental Health, The University of Melbourne, Parkville, 3010, Australia; Melbourne Bioinformatics, The University of Melbourne, Parkville, 3051, Australia; School of Biomedical Sciences and Pharmacy, University of Newcastle, Callaghan, 2308, Australia; Immune Health Program, Hunter Medical Research Institute, New Lambton Heights, 2305, Australia; Department of Neurology, John Hunter Hospital, New Lambton Heights, 2305, Australia; MS Research Flagship, Menzies Institute for Medical Research, University of Tasmania, Hobart, 7000, Australia; School of Biomedical Sciences and Pharmacy, University of Newcastle, Callaghan, 2308, Australia; Immune Health Program, Hunter Medical Research Institute, New Lambton Heights, 2305, Australia; Department of Neurology, John Hunter Hospital, New Lambton Heights, 2305, Australia; School of Biomedical Sciences and Pharmacy, University of Newcastle, Callaghan, 2308, Australia; Immune Health Program, Hunter Medical Research Institute, New Lambton Heights, 2305, Australia; School of Medicine and Dentistry, Gold Coast Campus, Griffith University, Southport, 4215, Australia; School of Biomedical Sciences and Pharmacy, University of Newcastle, Callaghan, 2308, Australia; Immune Health Program, Hunter Medical Research Institute, New Lambton Heights, 2305, Australia; Department of Clinical Neuroscience, Karolinska Institutet, Stockholm, 171 77, Sweden; Department of Clinical Neuroscience, Karolinska Institutet, Stockholm, 171 77, Sweden; Department of Clinical Neuroscience, Karolinska Institutet, Stockholm, 171 77, Sweden; Center for Molecular Medicine, Karolinska University Hospital, Stockholm, 171 76, Sweden; Institute of Environmental Medicine, Karolinska Institutet, Stockholm, 171 77, Sweden; Department of Clinical Neuroscience, Karolinska Institutet, Stockholm, 171 77, Sweden; Center for Molecular Medicine, Karolinska University Hospital, Stockholm, 171 76, Sweden; Institute of Environmental Medicine, Karolinska Institutet, Stockholm, 171 77, Sweden; MS Research Flagship, Menzies Institute for Medical Research, University of Tasmania, Hobart, 7000, Australia; The Florey Institute of Neuroscience and Mental Health, The University of Melbourne, Parkville, 3010, Australia; Murdoch Children’s Research Institute, The University of Melbourne, Parkville, 3052, Australia

**Keywords:** multiple sclerosis, DNA methylation, mediation, human herpesvirus-6, case–control

## Abstract

In Ausimmune, an Australian multicenter incident case–control study, Epstein–Barr virus (EBV)-related measures, including anti-EBNA antibodies and infectious mononucleosis, show multiple sclerosis (MS) associations mediated by DNA methylation (DNAm). Human herpesvirus-6 (HHV-6) DNA has also been linked to increased MS onset risk, though its mechanisms remain unknown. Therefore, we examined an expanded set of human herpesvirus indices including HHV-6 indices. We first tested associations with MS-associated DNAm modules, then assessed whether HHV-6 DNA contributes to MS onset through DNAm pathways. Serological (serum) and viral load (whole blood) measures of EBV (DNA, viral capsid antigen, early antigen diffuse and restricted), HHV-6 (DNA, IgM, IgG), cytomegalovirus (CMV) (IgG), and varicella zoster virus (DNA, IgG) were collected. DNAm was measured from whole blood (Illumina Infinium Human Methylation EPIC v1). DNAm-module (A1–A5) scores were derived using an epigenome-wide association study for MS onset risk and dimension-reduction methods. A total of 206 cases and 348 matched controls were analyzed. Multivariable linear regression demonstrated associations between HHV-6 DNA positivity and the A2-module, and between higher CMV IgG and the A4 module. Counterfactual mediation analysis indicated that 45% of the positive association of HHV-6 DNA positivity with MS onset risk was mediated through the A2 module (*P*_indirect_ = .008). The A2 module showed enrichment for lymphatic and immune pathways. These results provide evidence for a distinct DNAm module as a plausible mechanism underlying the associations of HHV-6 with MS onset. Importantly, these epigenetic pathways appear to mediate associations with human herpesviruses beyond EBV. These findings provide further insights into how environmental factors relate to MS onset through epigenetic programming.

## Introduction

Multiple sclerosis (MS) is an immune-mediated inflammatory condition of the central nervous system (CNS) characterized by inflammatory events that induce demyelination of neuronal axons. The exact pathogenesis of MS onset is unclear, but evidence supports a complex interplay between genetic and environmental factors, including human herpesviruses (HHVs) [1]. Among the HHVs implicated in MS onset, the strongest and most consistently replicated association is with Epstein–Barr virus (EBV) [2,3]. However, the contribution of other HHVs is less clear and remains uncertain.

Serological evidence of increased risk of MS with high titers of anti-EBV antibodies has already been described in the 1980s [4]. This association is recently further supported by findings from a large cohort study of millions of US military recruits monitored over a 20-year period [2]. In this study, individuals who were seronegative for EBV at baseline (young adulthood) but subsequently seroconverted exhibited a 32-fold greater risk of developing MS compared to those who remained seronegative, with seroconversion occurring prior to the onset of the disease. Using Ausimmune, we previously demonstrated that MS cases had higher titers of antibodies to EBV viral capsid antigen (VCA), and early antigen-restricted (EA-R) IgG, compared to matched controls; and that higher anti-EBNA titers and a history of infectious mononucleosis (IM) were associated with increased first clinical diagnosis (FCD) of CNS demyelination risk [5].

Other HHVs, including human herpesvirus 6 (HHV-6), cytomegalovirus (CMV), and varicella zoster virus (VZV), have also been implicated in MS. While there are two distinct species of HHV-6, HHV-6B and HHV-6A [6], many studies have assessed HHV-6 in aggregate, showing associations with higher MS risk [1, 7 , 8–11]. VZV has also been associated with increased risk of MS [7, 11 ,12]. In contrast to the positive associations seen for other HHVs and MS, CMV has generally been inversely associated with MS risk [1, 7, 13–18]. The mechanisms are unclear. In the Ausimmune study, we found that HHV-6 DNA positivity, extracted from whole blood and measured using real-time polymerase chain reaction, was associated with an increased risk of FCD. However, HHV-6 IgG or IgM, CMV IgG, VZV DNA positivity, and VZV IgG were not associated with FCD risk and no interactions between viral indices, including EBV, were reported [19].

Human leukocyte antigen (HLA) genes are well-established risk factors for MS. Interactions between HLA loci and HHVs have been reported to modify the risk of MS. These include additive interactions between high anti-EBNA antibodies, history of IM, and HLA-DRB1*1501, as well as interactions between high anti-EBNA and *HLA-A* single nucleotide polymorphisms (SNPs) [5]. Further, the genetic control of HHV-6A antibody responses has also been mapped to the HLA region [20]. These findings highlight the need to investigate the interplay between genetic risk factors and HHV exposure at a molecular level, providing insight into their combined impact on MS risk.

While there is evidence for EBV and other HHVs playing a role in MS risk, the underlying mechanism for these associations is unclear. Several have been proposed, such as molecular mimicry, in which immune cells cross-react with both exogenous antigens and CNS autoantigens [21] leading to activation of autoreactive T cells [22], or alternatively, an impaired T-cell responses to HHVs [23]. A better understanding of the molecular mechanisms underlying these relationships may provide further insights into how these viruses contribute to MS onset.

Epigenetic processes regulate gene expression without altering the underlying DNA sequence [24]. Of these, one of the most widely explored is DNA methylation (DNAm), which typically occurs at the 5-position of cytosine within a cytosine–guanine dinucleotide (CpG site) [25]. DNAm plays a fundamental role in modulating when (in time) and where (in the body) genes are expressed in response to genetic and environmental cues. With respect to MS, DNAm signatures have been identified that appear specific to this disease [26].

While epigenetic programming by DNAm is important in its own right, there is abundant evidence that it can mediate the effects of genetic and environmental risk factors on disease [27]. Accordingly, we recently showed that differential DNAm mediated large proportions of the associations between several canonical risk factors for MS, including self-reported and serological measures of EBV infection [28]. Although other HHVs have been implicated in MS onset, epigenetic mechanisms have not yet been investigated for their role in mediating this association. Further research is needed to determine whether there are specific epigenetic changes associated with HHVs and whether these changes play a role in MS disease development.

To this end, here we examined an expanded set of human herpesvirus indices, including those of HHV-6. We examined associations between these markers and MS-associated DNA methylation modules (DNAm modules) formed by weighted gene correlation network analysis (WGCNA), enabling network-level investigations of system-level epigenomic patterns [29]. We then conducted mediation analysis with a focus on examining whether HHV-6 DNA positivity influences MS onset risk via alterations in DNAm modules. Next, we examined whether the strength and direction of these effects vary according to HLA genotype. Finally, we assessed whether DNAm modules that mediate HHV–MS associations are enriched for immune-related pathways, relevant cell types, and transcriptional regulators.

## Results

The analysis was limited to cases (*n* = 206) who had converted to MS by 10-year follow-up, along with their matched controls (*n* = 348). Among the cases, 107 were first demyelinating event (FDE) with no prior undiagnosed episodes of suspected demyelination, 84 were FCD with suspected prior undiagnosed episodes, and 15 were progressive-onset cases. The median age for cases was 38.8 (IQR: 31.4–46.6) years and for controls 39.7 (IQR: 32.5–47.6) years. The study sample was 78% female (Table 1). Descriptive statistics for HHV serological and viral load indices can be found in Table 1. Consistent with previous reports, anti-VCA, anti-EA-R, and HHV-6 DNA positivity were each positively associated with MS risk (Table S2) [5, 19].

**Table 1.** Ausimmune analysis sample characteristics.^a^

	Cases	Cases: median	Controls	Controls: median	
Characteristic	(*N* = 206)	(IQR), *n* (%)	(*N* = 348)	(IQR), *n* (%)	*P-*value^b^
FCD onset type	206				
FDE		107 (51.9%)			
FCD with historical		84 (40.8%)			
Progressive-onset		15 (7.3%)			
Disease-modifying therapy use	206				
No		163 (79.1%)			
Yes		43 (20.9%)			
Age	206	38.80 (31.35–46.55)	348	39.71 (32.51–47.62)	.136
Sex (female vs male)	206	161 (78.2%)	348	273 (78.7%)	.936
Study region	206		347		.346
Brisbane City, QLD		75 (36.4%)		139 (40.1%)	
Newcastle city and surrounds, NSW		21 (10.2%)		35 (10.1%)	
Geelong city and the Western Districts, VIC		53 (25.7%)		100 (28.8%)	
State of Tasmania		57 (27.7%)		73 (21.0%)	
HHV-6 DNA load, copies/ml	165		162		*.030*
0		142 (86.1%)		154 (95.1%)	
200		19 (11.5%)		8 (4.9%)	
400		2 (1.2%)		0 (0.0%)	
>1 000 000		2 (1.2%)		0 (0.0%)	
Anti-HHV-6 IgM, dilutions/titers	161		154		.697
0		154 (95.7%)		144 (93.5%)	
20		4 (2.5%)		6 (3.9%)	
40		3 (1.9%)		4 (2.6%)	
Anti-HHV-6 IgG, dilutions/titers	164		160		.541
≤40		47 (28.7%)		55 (34.4%)	
160		79 (48.2%)		71 (44.4%)	
≥2500		38 (23.2%)		34 (21.2%)	
Anti-CMV IgG, optical density	165		162		.468
0		49 (29.7%)		38 (23.5%)	
<1		28 (17.0%)		26 (16.0%)	
1–10		38 (23.0%)		37 (22.8%)	
>10		50 (30.3%)		61 (37.7%)	
VZV-DNA load, copies/ml	165		162		.278
0		158 (95.8%)		149 (92.0%)	
200–1000		0 (0.0%)		3 (1.9%)	
1200–8200		4 (2.4%)		5 (3.1%)	
>10 200		3 (1.8%)		5 (3.1%)	
Anti-VZV IgG, optical density	165		162		.355
<0.5		11 (6.7%)		20 (12.3%)	
0.5 to <1.0		63 (38.2%)		57 (35.2%)	
1.0 to <1.5		82 (49.7%)		75 (46.3%)	
1.5–2		9 (5.5%)		10 (6.2%)	
EBNA DNA positivity	167		163		.280
Negative		149 (89.2%)		151 (92.6%)	
Positive		18 (10.8%)		12 (7.4%)	
Anti-VCA IgG antibody titer, dilutions/titers	165		162		.058
≤40		3 (1.8%)		11 (6.8%)	
160		30 (18.2%)		34 (21.0%)	
≥640		132 (80.0%)		117 (72.2%)	
EBV early antigen diffuse (anti-EA-D IgG), dilutions/titers	165		162		.867
≤40		144 (87.3%)		139 (85.8%)	
160		19 (11.5%)		20 (12.3%)	
≥640		2 (1.2%)		3 (1.9%)	
EBV early antigen restricted (anti-EA-R IgG), dilutions/titers	165		162		.059
≤40		110 (66.7%)		127 (78.4%)	
160		45 (27.3%)		29 (17.9%)	
≥640		10 (6.1%)		6 (3.7%)	
HLA-DRB1*1501, SNP rs3135388	206		346		*<0.001*
GG		91 (44.2%)		248 (71.7%)	
AA/AG*		115 (55.8%)		98 (28.3%)	
*HLA-A:02*, SNP rs2844821	206		346		*<0.001*
AG		73 (35.4%)		176 (50.9%)	
AA*		133 (64.6%)		170 (49.1%)	

Abbreviations: EBV, Epstein–Barr virus; VCA, viral capsid antigen; EA-R, early antigen restricted; EA-D, early antigen diffuse; HHV-6, human herpesvirus 6; CMV, cytomegalovirus; VZV, varicella zoster virus; IQR, interquartile range; OD, optical density.

a Analyses were limited to cases who had converted to clinically definite MS by 10-year follow-up and their matched controls, and that had DNAm data, *risk gene variant. HLA variables were coded as dichotomized factors to obtain odds ratios (ORs) > 1: HLA-DRB1*1501 SNP rs3135388 (AA/AG vs GG), HLA-A:02 SNP rs2844821 (AA vs AG), *risk gene variant.

b *P*-value for difference between cases and controls assessed using *t*-tests for continuous variables and χ^2^ or Fisher’s exact tests for categorical variables; italicized face indicates *P* < .05.

### HHV-6 DNA positivity and higher CMV IgG titer levels were associated with DNAm modules

**Figure 1:**
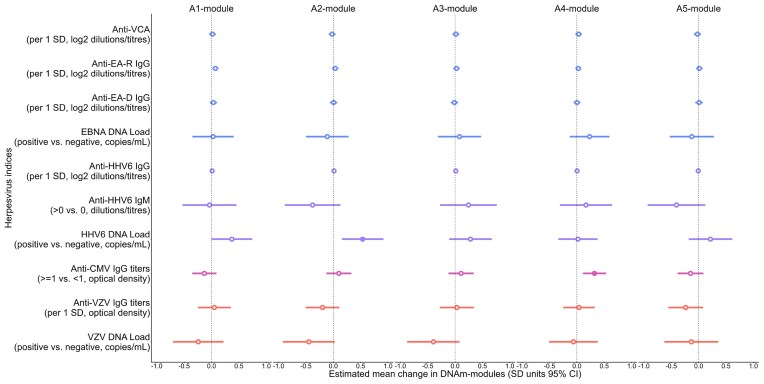
The associations between human herpesvirus indices and DNAm modules. Filled circles indicate significant q-values (adjusted using the Benjamini–Hochberg method). Adjusted for age, sex, and region. VCA, viral capsid antigen; EA-R, early antigen restricted; EA-D, early antigen diffuse; HHV-6, human herpesvirus 6; CMV, cytomegalovirus; VZV, varicella zoster virus. The A1 module included 436 CpGs across 407 genes; the A2 module included 687 CpGs across 627 genes; the A3 module included 74 CpGs across 70 genes; the A4 module included 29 CpGs across 29 genes; and the A5 module included 35 CpGs across 35 genes.

Two viral-module associations were observed. HHV-6 DNA positivity was associated with the A2 module (β = 0.02, 95% CI = 0.01–0.04, q = 0.050). CMV IgG titer was associated with the A4 module (β = 0.01, 95% CI = 0.01–0.02, q = 0.020). Anti-VCA IgG, anti-EA-R, anti-EA-D, HHV-6 IgG and IgM, VZV IgG, and VZV DNA positivity were not associated with any DNAm modules. Similarly, the A1, A3 and A5 modules showed no associations with any viral indices (Fig. 1; Table S3). These findings suggest that HHV-6 DNA positivity is associated with epigenetic variation.

### DNAm module mediates the relationships between HHV-6 DNA positivity and MS onset

**Figure 2:**
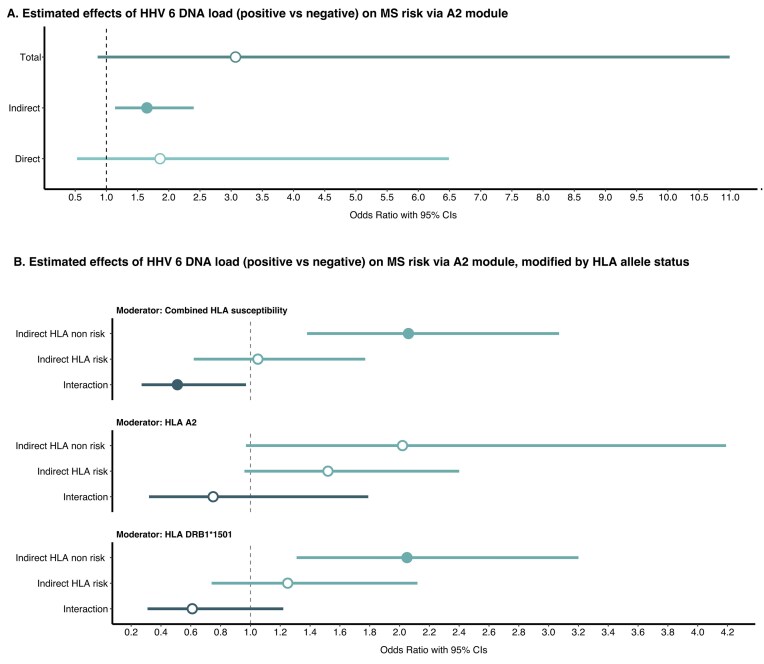
(A) The association between HHV-6 DNA positivity and multiple sclerosis with further consideration of the A2 module as a mediating factor. (B) The association between human HHV-6 DNA positivity and multiple sclerosis with further consideration of DNAm module as mediating factors, with mediation effects modified by HLA allele status. The analyses were adjusted for age, sex, and region. HLA variables were coded as dichotomized factors to obtain odds ratios (ORs) > 1: HLA-DRB1*1501 SNP rs3135388 (AA/AG vs GG), *HLA-A:02* SNP rs2844821 (AA vs AG). Combined HLA susceptibility = individuals with both HLA-DRB1*1501 AA/AG and *HLA-A:02* AA genotypes, susceptible vs all others, nonsusceptible. The A2 module included 687 CpGs across 627 genes.

Formal mediation analysis was conducted to evaluate the extent to which HHV-6 DNA positivity was operating through the A2 module to alter MS risk. The indirect effect estimate indicated that the A2 module mediated the association between HHV-6 DNA positivity and MS onset (OR_indirect_ = 1.65; 95% CI = 1.14–2.40, *P* = .008, Fig. 2; Table S4). The estimated proportion of the effect mediated was 45%. These results persisted after restricting to FDE cases (Table S5), excluding disease-modifying therapy (DMT)-treated cases (Table S6) and applying IPW (data not shown). Reverse causation testing demonstrated no significant mediation of case status to HHV-6 DNA positivity via the A2 module (Table S7). Previously, we found that the A2-module gene set was significantly enriched for GWAS MS risk genes [28]. Specifically, the top 200 genes in the A2 module contained 3.0 times more of the top 200 MS GWAS hits than expected by chance (hypergeometric *P* = .015), indicating that epigenetic changes may precede clinical onset, as the top module genes are genetically linked to MS. Among the A2 module CpGs set, 12.4% had at least one previously reported significant cis-meQTL in the EPIGEN MeQTL Database. These CpGs therefore have evidence of nearby genetic variants associated with variation in DNAm levels in peripheral blood.

HHV-6 DNA positivity was associated with estimated CD4T cell composition (Table S8). Cell-type-specific methylation PC1 scores derived using Tensor Composition Analysis (TCA) showed that mediation of the association between HHV-6 DNA positivity, and the MS risk was primarily observed in CD8T, CD4T, monocyte, and granulocyte compartments, with little evidence of contribution from natural killer (NK) or B-cell compartments (Table S9).

### DNAm mediation pattern varies across HLA genotypes

We next assessed whether the observed DNAm mediation effect differed by HLA-DRB1*1501 and *HLA-A:02* variants. The indirect effect for the association between HHV-6 DNA positivity and MS onset did not differ significantly by HLA-DRB1*1501 genotype (OR_interaction_ = 0.61; 95% CI = 0.31–1.22, *P* = .16), or by *HLA-A:02* genotype (OR_interaction_ = 0.75; 95% CI = 0.32–1.79, *P* = .519, Fig. 2B; Table S10). However, there was some evidence to indicate that the indirect effect differs between those with both the HLA-DRB1*1501 and *HLA-A:02* risk genotypes compared to those with all other combinations (OR_interaction_ = 0.51; 95% CI = 0.27–0.97, *P* = .041, Fig. 2B; Table S8). Thus, HHV-6 epigenetic mediation through the A2 module varied by MS risk HLA genotypes.

### Reactome and GO pathway enrichment characteristics of A2 module

**Figure 3:**
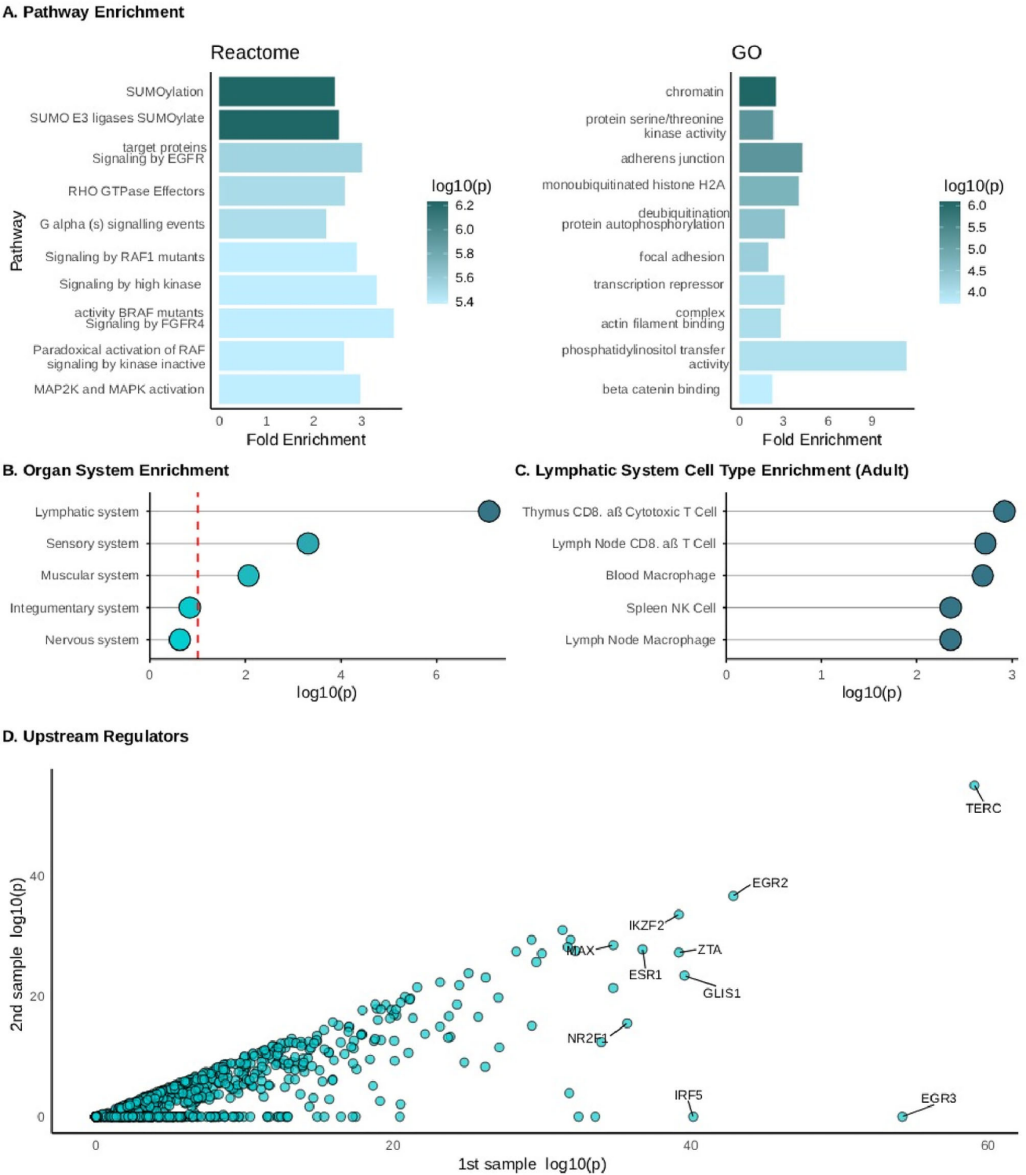
Enrichment analyses of A2 module 627 genes. (A) Reactome and gene ontology (GO) pathway enrichment analysis (top 10 pathways). (B) Enrichment of A2 module for adult cell-type marker genes, with *P*-values aggregated at the organ system level using Fisher’s method (top five organ systems) and (C) shown at the cell-type-specific level within adult lymphatic system tissue (top five cell types). (D) Upstream regulators of A2 module gene set inferred using the LISA platform. LISA provides *P-*values for each regulator across five ChIP-seq samples; the two lowest *P*-values are shown for each regulator. Because ranking is performed independently for each transcription factor, the first and second samples may correspond to different ChIP-seq experiments across transcription factors. Labeled are the top 10 of 1316 regulators tested in total.

As previously reported [28], the A2 module comprised 687 CpGs across 627 genes. This gene set was most enriched for the Reactome pathways *SUMO E3 ligates SUMOylate target proteins, SUMOylation*, and *RHO GTPase effectors* (Fig. 3A).

### Assessment of cell-type enrichment of A2-module gene set

Using WebCSEA, we investigated the adult tissue and cell types potentially affected by disruptions in the A2 module gene set. The A2 module was enriched for lymphatic system cell-type marker genes (*P* = 8.00 × 10^−8^) (Fig. 3B). The highest-ranked cell types were *thymus CD8+ αβ cytotoxic T cell* (*P* = 1.20 × 10^−3^), *lymph node CD8+ αβ T cell* (*P* = 1.90 × 10^−3^), *blood macrophage* (*P* = 2.03 × 10^−3^), and *spleen NK cell* (*P* = 4.40 × 10^−3^) (Fig. 3C). These results underscore the specific involvement of the immune system and its associated cell types in the A2 module.

### Inferred upstream transcription factors in the A2 module

To explore potential regulatory mechanisms linking the A2 module to MS, we identified the top 10 transcription factors associated with the A2 module (Fig. 3D), 4 of which have prior annotations to MS. These included EGR2, ESR1, IKZF2, and IRF5. We did not find any links between the targets of assessed antiviral medications (famciclovir, spironolactone, and tenofovir alafenamide) and the top 10 transcription factors for the A2 module.

### Supplementary analyses

Because causal mediation analysis can detect indirect effects even in the absence of a significant total effect [30], we examined all HHV DNAm-module associations for mediation. An additional mediation pathway involving anti-CMV IgG, the A4 module, and MS onset risk is presented in the Supplementary Analysis.

## Discussion

Here, we extended our past work on EBNA IgG, IM, and MS onset to further demonstrate that other human herpesviruses are associated with differential DNAm. There was evidence to indicate that HHV-6 DNA positivity and higher anti-CMV IgG titer levels were associated with DNAm changes captured by WGCNA-derived DNAm modules. We did not observe any associations between the DNAm modules and the other HHV markers assessed, including, anti-VCA IgG titer, anti-EA-R IgG, anti-EA-D IgG titer, EBV DNA positivity, anti-HHV-6 IgG titer, anti-HHV-6 IgM titer, anti-VZV IgG titer, and VZV DNA positivity. Mediation analysis demonstrated that the A2 module mediated almost half (45%) of the effect of HHV-6 DNA positivity on MS onset risk. This DNAm module is distinct from the A1 module, with which we previously found mediated EBV associations in the same study population [28]. The analyses indicated that HHV-6 DNA positivity is associated with specific DNAm changes not linked to EBV, indicating independent epigenetic influences related to MS risk.

### Immune associations reinforce A2 module’s role in HHV-6 infection and MS risk

The findings of the cell-specific mediation analysis suggested that the observed signal may be driven in part by methylation changes occurring within T-cell and myeloid compartments rather than NK or B-cell populations. To further assess biological plausibility, we investigated whether the A2 module captured signatures consistent with HHV-6-related immune pathways. The A2 module displayed strong enrichment for immune system cell-type marker genes. The strongest enrichment was for *thymus CD8^+^ αβ cytotoxic T cells*. The primary infection target for both HHV-6 species is the CD4^+^ T lymphocyte; however, HHV-6 has been shown to replicate in various cytotoxic effector cells including CD8^+^ T cells [31]. Further, cytotoxic T cells are an important part of the adaptive immune system. Specifically, they play a role in cell-mediated immunity, by recognizing viral peptides presented by MHC class I molecules on infected cells and then directly killing these cells [32]. These findings indicate that the A2 module may reflect epigenetic patterns associated with a T-cell immune response, though further work is needed to clarify the precise mechanism.

Additionally, upstream regulator analysis indicated TERC was the highest-ranked transcription factor. TERC has been reported to promote a cellular inflammatory response by stimulating the NK-κB pathway [33]. Another enriched transcription factor, IRF5, has been linked to HHV-6 replication and increased MS risk [34], and contributes to the pathogenesis of many inflammatory and autoimmune diseases [34,35]. Other enriched transcription factors ERG2 and ERG3 are reported to be involved in immune system regulation [36]. Lastly, pathway analysis showed that the A2 module gene set was enriched for genes involved in SUMOylation, a process that influences the production of interferon [37]. Together, these findings reinforce the immune-related nature of this module.

### Differing patterns of HHV markers with DNAm modules align with prior findings

While HHV-6 DNA positivity was associated with the A2 module, no corresponding associations were observed for HHV-6 IgM or IgG, precluding further mediation analysis. This is not unexpected, as neither of these antibody measures were previously associated with MS onset risk in the same study, and the IgM response was detectable in only a very small number of participants [19]. Failure to distinguish between HHV-6A and HHV-6B may also have contributed to the null serology findings [20].

Distinct markers reflect different phases of the HHV life cycle. Latent EBV infection is reflected by anti-EBNA IgG, anti-EA-R IgG can persist after primary infection reflecting prior lytic activity, acute infection/reactivation is reflected by anti-VCA IgG and anti-EA-D IgG, and ongoing viral replication is reflected by EBV DNA load [38]. We first examined exposure–mediator associations adjusted for multiple testing using the Benjamini–Hochberg procedure to reduce the likelihood of chance findings. As multiple of the exposures tested captured aspects of EBV infection and host response, this correction may have been overly stringent, and nominally significant associations remain biologically plausible. Notably, the association between anti-EA-R IgG and the A1 module showed directional consistency with both anti-EBNA IgG titer and a history of IM, as previously documented [28]. Further, our findings reinforce the importance of anti-EBNA IgG in MS risk. Previous work in this same study found that FCD risk linked to acute/reactivation antibodies (anti-VCA and anti-EA-D IgG) was no longer evident after adjusting for anti-EBNA IgG [5]; we found no association between these acute/reactivation antibodies and the A1 module. Taken together, this pattern is consistent with the A1 module being most strongly related to markers of past infection (anti-EBNA IgG, anti-EA-R IgG), with the observed association with IM aligning with clinically apparent primary infection, although further work is needed to substantiate these observations.

In evaluating mediation of the CMV IgG-MS associations by the A4 module, we identified a direct and an indirect effect with differing directions of association. The apparent protective direct effect is consistent with past work [14, 15 ,18]. Additionally, we observed a positive indirect effect. This indicates that CMV may act by both epigenetic and nonepigenetic pathways to alter MS onset risk, requiring additional method approaches as discussed in Supplementary Box 1. Further, the effect of CMV on progression, not only onset, should be considered. Effects may extend beyond disease onset, as elevated immune responses against CMV at disease onset have been associated with more favorable long-term disability outcomes [39]. Future work examining how DNAm pathways relate to disease progression, in addition to onset, will be important to understand the broader implications of CMV-associated epigenetic changes in MS.

### Temporality assessment of mediation findings enhances support of HHV associations with MS risk

We conducted several sensitivity analyses to examine the temporal order of the exposure, mediator, and outcome. Reverse-mediation analyses showed that the HHV-6 model was not significant. Enrichment of MS risk genes in the A2 module further supports the temporality sequence, suggesting that epigenetic changes in this module linked to environmental risk factors may precede clinical onset. Additionally, a sensitivity analysis limiting to treatment-naïve cases did not change the results. This limits the potential for differences in the cases by DMT status, differences that include modulation of the immune/inflammatory state by which presumably viruses act to impact disease. We applied IPW, and results were unchanged, suggesting that selection bias is unlikely to explain the observed effects. Taken together, the consistency of results across bias analyses increases confidence in our primary mediation findings.

### HLA status influences the role of DNAm pathways in MS risk

Individuals without the MS risk HLA alleles showed stronger reliance on the methylation-mediated pathway. The effect in those with the MS risk alleles was less dependent on this pathway, possibly suggesting the involvement of additional mechanisms. Furthermore, the two groups may have different baseline methylation profiles, potentially leading to different immune system priming thresholds as these alleles are receptors for viral infection. These findings highlight the need to understand the interplay between herpesvirus, associated genetic variants, and epigenetic changes, rather than considering each separately.

### Strengths

Our results are in keeping with a number of the Bradford Hill criteria for causal inference [40], including strength, biological plausibility, and specificity. The mediation analysis demonstrates a significant indirect effect, with 45% of the association between HHV-6 DNA positivity and MS onset risk mediated by the A2 module. This indicates a strong link between exposure and outcome, reinforcing the strength of association criteria. Biological plausibility is underscored by the enrichment of the A2 module for immune system cell-type marker genes and immune-related transcription factors and pathways. The results are consistent with previous research implicating HHV involvement in MS onset and further builds on this by exploring epigenetic mechanisms that are coherent with the current biological understanding, providing evidence that environmental factors influence MS pathogenesis via immune-related pathways.

### Limitations

Limitations include that we were unable to differentiate between HHV-6 A and HHV-6B. This was due to the methods to assay HHV6-type-specific serology not being available to do so when the serology work was undertaken. Therefore, further work should examine the observed HHV-6 results for only HHV-6A serology/viral load. The high HHV-6 DNA load observed in two of the cases could represent chromosomal integration; however, we did not have enough samples available to confirm this. A sensitivity analysis where these two participants were excluded did not change the results (Supplementary Analysis). Therefore, the observed results are not driven only by the potential cases of chromosomal integration. However, future studies with the ability to confirm chromosomal integration would be valuable to validate these results. Although the biological plausibility of the results supports their credibility, validation of these findings through independent prospective cohorts with similar viral exposure and epigenetic data would be valuable. Lastly, our analysis was conducted using whole blood. Future studies examining DNAm within individual cell populations would therefore be valuable, extending the TCA analysis presented here.

## Conclusion

Overall, our study indicates associations between HHVs, beyond EBV, and MS onset risk, potentially involving epigenetic profiles. Specifically, we observed associations with the A2 module for HHV-6 DNA positivity. These results also provide evidence that the HHVs considered here are not all operating through common epigenetic mechanisms and as such future work needs to consider each virus independently. These findings provide further insights into herpesvirus effect on MS onset.

## Material and methods

### Study population

The Ausimmune study has previously been described in detail [41]. Cases comprised patients aged 18–59 years who were referred to the study by neurologists and radiologists after presenting with symptoms diagnosed as a first clinical demyelination (FCD). For this analysis, the inclusion criteria for MS cases were: (1) FCD cases who did not have a non-MS diagnosis by 10-year follow-up (McDonald 2001 criteria), (2) serological and viral DNA samples available, and (3) DNAm data available. FCD cases were classified by a neurologist as: “first demyelinating event (FDE)” with no prior undiagnosed episodes of suspected demyelination, FCD with suspected prior undiagnosed episodes, and progressive-onset cases. Control participants were randomly selected from the Australian electoral roll and matched to cases by age (within two years), sex, and study region [41]. We utilized matched controls with DNAm data available. Ausimmune was approved by nine regional human research ethics committees and all participants provided written informed consent.

### Measurement of HHV indices

Viral biomarkers were measured in whole blood and serum collected at Ausimmune baseline interview. Inclusion in these analyses was based on availability of a biological sample for cases and at least one matched control. Further details of the viral biomarker substudy and measurement of samples have been previously reported [19]. Briefly, whole blood and serum samples were obtained and stored in 1 ml aliquots at −80°C until analysis. Viral DNA was extracted from whole blood using quantitative real-time polymerase chain reaction, reported as copies/ml. The EBNA-1 gene region was targeted for EBV DNA load. The U67 gene was targeted for HHV-6 DNA load and the VZV IE gene was targeted for VZV DNA load. From serum, quantitative IgG antibody titers to EBV VCA were measured by automated enzyme immunoassay (Star Corp, Stillwater, MN). Immunofluorescence assays were used to measure antibodies to the EBNA complex, early antigen diffuse (EA-D), early antigen restricted (EA-R), and HHV-6 (both IgG and IgM), with results expressed as dilutions/titers. Similarly, immunofluorescence assays were used to measure anti-CMV IgG and anti-VZV IgG; these results were expressed as optical density values.

As in our previous reports, viral DNA for all tested viruses was detectable in ~50% of samples, and among those, levels were highly skewed. As such binary variables (positive/negative) were used in all viral DNA analyses [5,19]. Anti-VCA IgG, anti-EA-D IgG, anti-EA-R IgG, and anti-HHV-6 IgG were modeled as continuous variables using a log base 2 of the reciprocal of the dilution measured as titer transformation. Anti-VZV IgG was modeled as a continuous (nontransformed) variable. Anti-HHV-6 IgM was modeled as a binary (>0/0) variable because very few participants had measurable (nonzero) values, particularly when split by case–control status. Anti-CMV IgG levels were modeled as binary (low/high) due to high skew and no suitable transformation [5,19].

### Genotype and DNA methylation measurement

Details of the genome-wide genotyping and DNAm measures have been previously described [26]. Briefly, DNA was extracted from whole blood samples collected at baseline using QIAamp DNA Blood Mini kit^TM^ (Qiagen, Netherlands). To determine SNP profiles, genotyping for *HLA-A:02* (SNP rs2844821 on Illumina Custom MS Chip) was performed by the Hussman Institute for Human Genomics, University of Miami (USA) [42]. The Global Screening Array v2.1 (Illumina, USA) was used according to the manufacturer’s guidelines to determine HLA-DRB1*15:01 haplotype (SNP rs3135388) [26].

DNAm was assayed using the Illumina Infinium Human Methylation EPIC v1 BeadChip Kit (Illumina, USA).

### Other measures

As described previously [41], participants completed a comprehensive set of surveys querying a broad range of sociodemographic and environmental/lifestyle parameters. From this self-reported history of IM, age, sex, and residential region at recruitment was recorded. Participants were also assessed by a study nurse, who recorded medication use, including disease-modifying therapies (DMTs).

### Statistical methods

#### DNAm-module construction

The analysis was focused on system-level epigenomics [29, 43]. DNAm modules, which are MS-associated networks of CpGs, were constructed using WGCNA. To ensure MS-specific DNAm results, CpGs were first ranked according to their association with MS onset risk in an epigenome-wide association study (EWAS) implemented using a Bioconductor workflow. The EWAS model was adjusted for estimated cell-type proportions using the EpiDISH R package, including CD8⁺ T cells (CD8T), CD4⁺ T cells (CD4T), NK cells, B lymphocytes (Bcell), monocytes (Mono), and granulocytes (Gran), as well as ancestry (first two principal components) and study matching covariates. CpGs showing the strongest associations with MS risk (*n* = 2 432; Benjamini–Hochberg adjusted *P* < 5 × 10⁻⁶) were retained for downstream analysis. These CpGs were then used as input for WGCNA to identify clusters of co-methylated CpGs forming DNAm modules. WGCNA was implemented in R using standard parameters (unsigned network, minimum module size = 30, reassignment threshold = 0.05, merge cut height = 0.25), with scale-independence and mean connectivity assessments indicating an optimal soft-thresholding power of 7. This procedure resulted in five MS-associated DNAm modules (A1–A5), each summarized by its first principal component (module eigenCpG), which captures the overall methylation profile of the module for each participant.

#### Exposure–outcome associations

Exposure–outcome associations were assessed by multivariable logistic regression, adjusting for age, sex, and residential region at recruitment.

#### Evaluations of DNAm-module associations with HHV indices

Separate multivariable regression models were used to estimate associations between HHV indices and each of the five DNAm modules. The models were adjusted for sex, age, and residential region at recruitment. The results were corrected for multiple comparisons using the Benjamini–Hochberg method with q-values reported [44]. The q-value reflects the false discovery rate (FDR), and findings with q < 0.05 were considered significant, indicating a low likelihood of being false positives.

#### Mediation analyses of exposure–MS associations

In our prior analysis, all five DNAm modules were positively associated with risk of MS onset (Table S1) [28]. Counterfactual mediation analysis was used to evaluate mediators of exposure–MS associations. Here, the total effect (exposure–outcome) of HHV exposure on MS onset risk was decomposed into two components. The first component was the natural direct effect, representing the proportion of the effect that was not mediated by the DNAm module. The second component was the natural indirect effect, representing the proportion of the effect that was mediated by the DNAm module [45]. When a significant indirect effect was observed and the total, direct, and indirect effects were in the same direction, we calculated the proportion mediated as ln(OR_indirect_)/ln(OR_total_). Mediation was considered for any HHV index associated with a DNAm module, reflecting that causal mediation analysis can detect indirect effects even when a significant total effect is not present [30]. Significance level of 0.05 was applied in all mediation analyses. Mediation analyses were implemented using the medflex R package [46].

#### Sensitivity analyses

To reinforce the temporal sequence of exposure–mediator–outcome, we (i) limited the mediation analyses to FDEs without a prior history of suspected demyelination, (ii) evaluated whether the modules were enriched for MS risk genes using GWAS Catalog metadata (https://www.ebi.ac.uk/gwas/), and (iii) examined reverse-pathway mediation models (case status to mediator to exposure) to determine if the size and significance of effects were comparable to those observed in the primary mediation analyses.

To assess whether genetic variation could influence methylation at the CpGs identified in our study, we queried the EPIGEN MeQTL Database, a published blood meQTL resource generated using the Illumina Infinium MethylationEPIC array in 2358 samples from three UK cohorts [47]. Our list of module CpGs was intersected with the meQTL summary statistics, and CpGs were classified as having a previously reported cis-meQTL if at least one SNP within 1 Mbp showed a significant association with methylation (FDR < 0.05) in the published dataset [47].

To address any potential selection bias between participants with and without DNAm data, we applied inverse probability weighting (IPW), adjusting for age, sex, region, education, and ancestry (first two principal components) [26]. Additional analyses were conducted limiting to cases not on DMTs (*n* = 163).

To determine if mediated effects were cell-type specific, we first tested whether the viral indices were associated with cell-type composition, using multivariable linear regression adjusting for matching criteria. We then derived cell-type-specific methylation estimates using the TCA R package [48]. Here, the bulk DNA methylation beta values for CpGs within each module were used as the input matrix, along with estimated cell-type proportions. The resulting output provided separate methylation matrices for the six cell populations. These matrices were then filtered to the CpG set for each of the five DNAm modules and PCA was then performed. The first principal component (PC1) was extracted as a module summary score for each individual. These PC1 variables were subsequently evaluated as mediators in the mediation analyses.

To investigate whether observed indirect effects identified in the primary mediation analysis differed by HLA allele status, HLA variables were coded as dichotomized factors to obtain odds ratios (ORs) > 1: HLA-DRB1*1501 SNP rs3135388 (AA/AG vs GG), *HLA-A:02* SNP rs2844821 (AA vs AG). We conducted mediation analysis allowing indirect effect estimates to vary by three genetic risk profiles: HLA-DRB1*1501, HLA-A:02, and their combined status (extremes of exposure, individuals with both HLA-DRB1*1501 AA/AG and *HLA-A:02* AA genotypes). We fitted mediation models where the indirect effects were allowed to vary according to HLA allele status through product terms. The following parameters were estimated: the direct effect, the indirect effect for the reference HLA status (non-risk HLA allele), the indirect effect for the risk HLA allele, the interaction term quantifying how much the indirect effect differs in individuals with the MS risk HLA allele status compared to the nonrisk reference group and the total effects for each HLA group. Significant interaction terms would indicate that HLA allele status modifies the strength of the mediation pathway.

#### DNAm-module gene set enrichment analysis

To examine the biological relevance of any DNAm module found to mediate the HHV-MS onset associations, CpG sites within that module were mapped to their nearest gene to create gene sets.

The pathfindR algorithm, which performs active subnetwork enrichment analysis, was used to identify enriched pathways for each module gene set, using the Reactome and Gene Ontology databases as references. Benjamini–Hochberg adjustment was applied for multiple comparisons. A minimum overlap of two genes was used to ensure that pathway enrichment signals were supported by multiple genes within the module while maintaining sensitivity for smaller gene sets.

WebCSEA: web-based cell-type-specific enrichment analysis of genes was used to examine gene set enrichment among a collection of human tissue-cell-type expression signatures [49]. WebCSEA uses validated cell-type marker gene sets identified from bulk and single-cell RNA sequencing studies across multiple human tissues. Cell-type-specific *P*-values from WebCSEA were aggregated for each organ system using Fisher’s method. The resulting values were corrected for multiple testing with the Benjamini–Hochberg procedure and used to rank the organ systems.

The LISA platform [50] was used to infer the upstream regulators of the DNAm modules in silico. The default background gene set was used in the LISA analysis. LISA reports up to five Chip-seq samples for each transcriptional factor, after evaluating all available samples. The ChIP-seq sample reported therefore represents the most predictive ChIP-seq dataset for each transcription factor, noting that the specific datasets differ between transcription factors. The transcription factors are ranked by their statistical significance across chromatin accessibility and binding models [50]. Using the Open Targets platform [51], the 10 top-ranked transcription factors were then cross-referenced for reported associations with MS and with the antiviral medications famciclovir, spironolactone, and tenofovir alafenamide. These were selected as a scientific and lived experience expert panel has identified these medications as potential treatments to be used in a phase III clinical trial for MS [52].

#### Replication analysis

Analyses was undertaken for A3–A5 modules, as we previously demonstrated that the biological relevance of the A1 and A2 modules showed substantial overlap with modules in the Epidemiological Investigation of Multiple Sclerosis (EIMS) study [28]. Detailed methods are provided in Supplementary Analysis.

Statistical analyses were performed using R version 4.4.1 (R Foundation for Statistical Computing). All analyses were complete case.

## Supplementary Material

dvag016_Supplemental_File

## Data Availability

The Ausimmune and AusLong data used in this paper are available under restricted access for participant privacy. Requests for data access should be directed to the corresponding author, Professor Anne-Louise Ponsonby (annelouise.ponsonby@florey.edu.au). Requests will be considered by the Ausimmune/AusLong Steering Committee on scientific and ethical grounds and, if approved, provided under collaborative research agreements.

## Notes

The full list of people in the Ausimmune Investigators Group is included in the Acknowledgements.
