# Supplementary material for: DNA methylation mediates the multiple sclerosis onset risk associated with HHV-6 DNA positivity
Source: Environ Epigenet. 2026 May 25;12(1):dvag016. doi: 10.1093/eep/dvag016 (PMC13199913; doi:10.1093/eep/dvag016)
Supplement: dvag016_Supplemental_File [file dvag016_supplemental_file.docx]

# Supplementary materials

**DNA methylation mediates the multiple sclerosis onset risk associated with HHV-6 DNA positivity**

Alex Eisner, Steve Simpson-Yap, Daniel J Park, Ellen Morwitch, Samuel Tanner, Vicki E. Maltby, Ingrid van der Mei, Jeannette Lechner-Scott, Rodney J Scott, Simon A Broadley, Rod A Lea, Pernilla Stridh, Tomas Olsson, Maja Jagodic, Lars Alfredsson, Bruce V Taylor, Ausimmune Investigators Group, Anne-Louise Ponsonby

Contents

[Supplementary materials 1](#_Toc228536174)

[**Table S1.** Associations between DNAm-modules and MS onset 2](#_Toc228536175)

[**Table S2.** Associations of viral markers with MS onset 2](#_Toc228536176)

[**Table S3.** Associations between viral markers with DNAm-modules 3](#_Toc228536177)

[**Table S4.** The associations between human herpesvirus indices and multiple sclerosis with further consideration of the A2- and A4-module as mediating factors. 5](#_Toc228536178)

[**Table S5.** The associations between human herpesvirus indices and multiple sclerosis with further consideration of the A2-module as a mediating factor, restricted to FDEs. 5](#_Toc228536179)

[**Table S6.** The associations between human herpesvirus indices and multiple sclerosis with further consideration of the A2-module as a mediating factor, restricted to cases not being treated with disease-modifying therapies (DMTs) at time of survey. 6](#_Toc228536180)

[**Table S7.** Sensitivity analysis, reverse (MS-to-risk factor) mediation. 6](#_Toc228536181)

[**Table S8.** Association between HHV-6 DNA positivity, anti-CMV IgG and estimated blood cell-type composition. 7](#_Toc228536182)

[**Table S9.** Associations between viral indices and multiple sclerosis risk: evaluation of DNAm-modules as mediators across immune cell types. 8](#_Toc228536183)

[**Table S10.** The associations between human herpesvirus indices and multiple sclerosis with further consideration of the A2-module as a mediating factor, with mediation effects modified by HLA allele status. 10](#_Toc228536184)

[**Supplementary Analysis** 11](#_Toc228536185)

[**Supplementary Box 1. Investigation approaches to assess the role of the A4-module in CMV infection and MS risk.** 12](#_Toc228536186)

[**Figure S1.** Upstream regulators of A4-module gene set inferred using the LISA platform. The A4-module included 29 CpGs across 29 genes. Because ranking is performed independently for each transcription factor, the 1st and 2nd samples may correspond to different ChIP-seq experiments across transcription factors. 13](#_Toc228536187)

[**Figure S2.** Distribution of p-values for the association between randomised A4-module and MS onset risk. 14](#_Toc228536188)

## **Table S1.** Associations between DNAm-modules and MS onset

| **Module** | **AOR (95%CI)** | **P-value** |
| --- | --- | --- |
| *A1-module* | **6.00x10^14^ (9.37x10^11^, 6.57x10^17^)** | **3.30x10^-23^** |
| *A2-module* | **1.68x10^12^ (4.79x10^9^, 9.3x10^14^)** | **1.13x10^-19^** |
| *A3-module* | **2.85x10^7^ (2.55x10^5^, 4.10x10^9^)** | **3.45x10^-12^** |
| *A4-module* | **6.50x10^6^ (6.02x10^4^, 8.77x10^8^)** | **1.32x10^-10^** |
| *A5-module* | **1.48x10^6^ (1.67x10^4^, 1.59x10^8^)** | **1.13x10^-9^** |
| Associations between DNAm-modules and MS onset as reported by Simpson-Yap et al^13^. Models adjusted for age, sex, and study region. Results in boldface denote statistical significance (p<0.05). The A1‐module included 436 CpGs across 407 genes; The A2‐module included 687 CpGs across 627 genes; the A3-module included 74 CpGs across 70 genes; the A4-module included 29 CpGs across 29 genes; and the A5-module included 35 CpGs across 35 genes. | | |

## **Table S2.** Associations of viral markers with MS onset

|  | **AOR (95%CI)** | **P-value** |
| --- | --- | --- |
| EBV DNA (positive vs negative) | 1.53 (0.72, 3.39) | 0.276 |
| **VCA IgG (per log 2 increase)** | **1.17 (1.03, 1.33)** | **0.017** |
| **EA-R (per log 2 increase)** | **1.16 (1.03, 1.30)** | **0.010** |
| EA-D (per log 2 increase) | 1.06 (0.94, 1.22) | 0.315 |
| **HHV-6 DNA (positive vs negative)** | **3.09 (1.38, 7.62)** | **0.009** |
| HHV-6 IgG (per log 2 increase) | 1.04 (0.96, 1.13 | 0.356 |
| HHV-6 IgM (positive vs negative) | 0.63 (0.22, 1.71) | 0.368 |
| CMV IgG (positive vs negative) | 0.75 (0.48, 1.17) | 0.201 |
| VZV DNA (positive vs negative) | 0.48 (0.17, 1.24) | 0.140 |
| VZV IgG | 1.40 (0.77, 2.56) | 0.272 |

Abbreviations: EBV, Epstein Barr Virus; VCA, viral capsid antigen; EA-R, early antigen restricted; EA-D, early antigen diffuse; HHV-6, human herpesvirus 6; CMV, cytomegalovirus; VZV, varicella zoster virus. Models adjusted for age, sex, and study region. Results in boldface denote statistical significance (p<0.05).

## **Table S3.** Associations between viral markers with DNAm-modules

| **Exposure** | **Outcome** | **Beta (95% CI)** | **P-value** | **Q values** |
| --- | --- | --- | --- | --- |
| EA-R (per log 2 increase) | A1-module | 0.006 (0.001, 0.011) | 0.011 | 0.11 |
| HHV-6 DNA (positive vs negative) | A1-module | 0.016 (0.000, 0.031) | 0.046 | 0.23 |
| VZV DNA (positive vs negative) | A1-module | -0.010 (-0.030, 0.009) | 0.295 | 0.59 |
| CMV IgG (≥1 vs <1) | A1-module | -0.006 (-0.015, 0.004) | 0.238 | 0.59 |
| EA-D (per log 2 increase) | A1-module | 0.003 (-0.002, 0.007) | 0.294 | 0.59 |
| HHV-6 IgG (per log 2 increase) | A1-module | 0.001 (-0.001, 0.002) | 0.553 | 0.807 |
| VCA IgG (per log 2 increase) | A1-module | 0.001 (-0.003, 0.006) | 0.565 | 0.807 |
| VZV IgG | A1-module | 0.002 (-0.01, 0.015) | 0.721 | 0.880 |
| HHV-6 IgM (>0 vs 0) | A1-module | -0.002 (-0.022, 0.019) | 0.88 | 0.88 |
| EBV DNA (positive vs negative) | A1-module | 0.001 (-0.015, 0.017) | 0.878 | 0.88 |
| HHV-6 DNA (positive vs negative) | A2-module | **0.023 (0.007, 0.039)** | **0.005** | **0.05** |
| VZV DNA (positive vs negative) | A2-module | -0.019 (-0.039, 0.001) | 0.06 | 0.3 |
| HHV-6 IgM (>0 vs 0) | A2-module | -0.016 (-0.037, 0.005) | 0.137 | 0.343 |
| VZV IgG | A2-module | -0.008 (-0.021, 0.005) | 0.202 | 0.450 |
| HHV-6 IgG (per log 2 increase) | A2-module | 0.001 (-0.001, 0.002) | 0.521 | 0.636 |
| EA-R (per log 2 increase) | A2-module | 0.003 (-0.002, 0.008) | 0.225 | 0.375 |
| CMV IgG (≥1 vs <1) | A2-module | 0.004 (-0.005, 0.014) | 0.373 | 0.533 |
| VCA IgG (per log 2 increase) | A2-module | -0.002 (-0.006, 0.003) | 0.496 | 0.62 |
| EBV DNA (positive vs negative) | A2-module | -0.005 (-0.021, 0.012) | 0.572 | 0.636 |
| EA-D (per log 2 increase) | A2-module | 0.0004 (-0.004, 0.005) | 0.878 | 0.878 |
| HHV-6 DNA (positive vs negative) | A3-module | 0.012 (-0.004, 0.028) | 0.15 | 0.734 |
| HHV-6 IgM (>0 vs 0) | A3-module | 0.010 (-0.011, 0.032) | 0.346 | 0.734 |
| VZV DNA (positive vs negative) | A3-module | -0.017 (-0.037, 0.003) | 0.098 | 0.734 |
| CMV IgG (≥1 vs <1) | A3-module | 0.005 (-0.005, 0.014) | 0.341 | 0.734 |
| EA-R (per log 2 increase) | A3-module | 0.002 (-0.003, 0.007) | 0.367 | 0.734 |
| HHV-6 IgG (per log 2 increase) | A3-module | 0.001 (-0.001, 0.002) | 0.551 | 0.789 |
| VZV IgG | A3-module | 0.001 (-0.012, 0.014) | 0.837 | 0.837 |
| EA-D (per log 2 increase) | A3-module | -0.001 (-0.006, 0.004) | 0.71 | 0.771 |
| EBV DNA (positive vs negative) | A3-module | 0.003 (-0.013, 0.020) | 0.683 | 0.771 |
| VCA IgG (per log 2 increase) | A3-module | 0.001 (-0.004, 0.006) | 0.667 | 0.771 |
| CMV IgG (≥1 vs <1) | A4-module | **0.014 (0.005, 0.022)** | **0.002** | **0.02** |
| EA-R (per log 2 increase) | A4-module | 0.002 (-0.002, 0.007) | 0.309 | 0.773 |
| EBV DNA (positive vs negative) | A4-module | 0.010 (-0.005, 0.025) | 0.199 | 0.773 |
| VCA IgG (per log 2 increase) | A4-module | 0.002 (-0.002, 0.007) | 0.274 | 0.773 |
| HHV-6 IgM (>0 vs 0) | A4-module | 0.007 (-0.013, 0.027) | 0.484 | 0.968 |
| HHV-6 DNA (positive vs negative) | A4-module | 0.001 (-0.014, 0.016) | 0.913 | 0.969 |
| HHV-6 IgG (per log 2 increase) | A4-module | 0 (-0.001, 0.002) | 0.856 | 0.925 |
| VZV DNA (positive vs negative) | A4-module | -0.003 (-0.021, 0.016) | 0.778 | 0.969 |
| VZV IgG | A4-module | 0.002 (-0.01, 0.014) | 0.781 | 0.925 |
| EA-D (per log 2 increase) | A4-module | 0.0002 (-0.004, 0.005) | 0.925 | 0.969 |
| HHV-6 IgM (>0 vs 0) | A5-module | -0.017 (-0.039, 0.005) | 0.124 | 0.69 |
| VZV IgG | A5-module | -0.01 (-0.023, 0.003) | 0.137 | 0.685 |
| CMV IgG (≥1 vs <1) | A5-module | -0.006 (-0.016, 0.004) | 0.207 | 0.69 |
| HHV-6 DNA (positive vs negative) | A5-module | 0.009 (-0.007, 0.026) | 0.279 | 0.698 |
| HHV-6 IgG (per log 2 increase) | A5-module | 0 (-0.002, 0.001) | 0.733 | 0.814 |
| VZV DNA (positive vs negative) | A5-module | -0.006 (-0.026, 0.015) | 0.589 | 0.733 |
| EA-R (per log 2 increase) | A5-module | 0.001 (-0.004, 0.006) | 0.66 | 0.733 |
| EBV DNA (positive vs negative) | A5-module | -0.005 (-0.022, 0.011) | 0.532 | 0.733 |
| VCA IgG (per log 2 increase) | A5-module | -0.002 (-0.007, 0.003) | 0.482 | 0.733 |
| EA-D (per log 2 increase) | A5-module | 0.001 (-0.004, 0.006) | 0.823 | 0.823 |

Abbreviations: EBV, Epstein Barr Virus; VCA, viral capsid antigen; EA-R, early antigen restricted; EA-D, early antigen diffuse; HHV-6, human herpesvirus 6; CMV, cytomegalovirus; VZV, varicella zoster virus. Analysis adjusted for age, sex, and region. P-values were adjusted for multiple testing using the Benjamini–Hochberg procedure, and results are reported as q-values. Statistical significance was defined as q<0.05. The A1‐module included 436 CpGs across 407 genes; The A2‐module included 687 CpGs across 627 genes; the A3-module included 74 CpGs across 70 genes; the A4-module included 29 CpGs across 29 genes; and the A5-module included 35 CpGs across 35 genes.

## **Table S4.** The associations between human herpesvirus indices and multiple sclerosis with further consideration of the A2- and A4-module as mediating factors.

| **Exposure** | **Mediator** | **Outcome** | **Total effect** | | **Direct effect** | | **Indirect effect** | | % mediated |
| --- | --- | --- | --- | --- | --- | --- | --- | --- | --- |
|  |  |  | AOR (95%CI) | P-value | AOR (95%CI) | P-value | AOR (95%CI) | P-value |  |
| HHV-6 DNA (positive vs negative), | A2-module | MS onset | 3.07 (0.86, 10.99) | 0.084 | 1.86 (0.53, 6.49) | 0.33 | **1.65 (1.14, 2.4)** | **0.008** | 45% |
| CMV IgG (≥1 vs <1) | A4-module | MS onset | 0.76 (0.48, 1.2) | 0.24 | **0.62 (0.4, 0.96)** | **0.033** | **1.22 (1.04, 1.43)** | **0.013** | ─ |
|  |  |  |  |  |  |  |  |  |  |
| The analysis is adjusted for age, sex, and region. Percentage mediated only calculated when direct and indirect effect estimates are in the same direction, ─ indicates proportion mediated could not be calculated. HHV-6, human herpesvirus 6; CMV, cytomegalovirus. Results in boldface denote statistical significance (p<0.05). The A2‐module included 687 CpGs across 627 genes; the A4-module included 29 CpGs across 29 genes. | | | | | | | | | |

## **Table S5.** The associations between human herpesvirus indices and multiple sclerosis with further consideration of the A2-module as a mediating factor, restricted to FDEs.

| **Exposure** | **Mediator** | **Outcome** | **Total effect** | | **Direct effect** | | **Indirect effect** | | % mediated |
| --- | --- | --- | --- | --- | --- | --- | --- | --- | --- |
|  |  |  | AOR (95%CI) | P-value | AOR (95%CI) | P-value | AOR (95%CI) | P-value |  |
| HHV-6 DNA (positive vs negative) | A2-module | MS onset | 2.87 (0.93, 8.82) | 0.066 | 1.75 (0.57, 5.32) | 0.33 | **1.64 (1.03, 2.63)** | **0.039** | 47% |
| The analysis is adjusted for age, sex, and region. Percentage mediated only calculated when direct and indirect effect estimates are in the same direction. FDE, first demyelinating event; HHV-6, human herpesvirus 6. Results in boldface denote statistical significance (p<0.05). The A2‐module included 687 CpGs across 627 genes. | | | | | | | | | |

## **Table S6.** The associations between human herpesvirus indices and multiple sclerosis with further consideration of the A2-module as a mediating factor, restricted to cases not being treated with disease-modifying therapies (DMTs) at time of survey.

| **Exposure** | **Mediator** | **Outcome** | **Total effect** | | **Direct effect** | | **Indirect effect** | | % mediated |
| --- | --- | --- | --- | --- | --- | --- | --- | --- | --- |
|  |  |  | AOR (95%CI) | P-value | AOR (95%CI) | P-value | AOR (95%CI) | P-value |  |
| HHV-6 DNA (positive vs negative) | A2-module | MS onset | 2.61 (0.66, 10.25) | 0.17 | 1.43 (0.37, 5.49) | 0.6 | **1.82 (1.16, 2.85)** | **0.009** | 62% |
| The analysis is adjusted for age, sex, and region. Percentage mediated only calculated when direct and indirect effect estimates are in the same direction. HHV-6, human herpesvirus 6. Results in boldface denote statistical significance (p<0.05). The A2‐module included 687 CpGs across 627 genes. | | | | | | | | | |

## **Table S7.** Sensitivity analysis, reverse (MS-to-risk factor) mediation.

| **Exposure** | **Mediator** | **Outcome** | **Total effect** | | **Direct effect** | | **Indirect effect** | | % mediated |
| --- | --- | --- | --- | --- | --- | --- | --- | --- | --- |
|  |  |  | AOR (95%CI) | P-value | AOR (95%CI) | P-value | AOR (95%CI) | P-value |  |
| MS onset | A2-module | HHV-6 DNA (positive vs negative) | 3.09 (0.56, 17.03) | 0.200 | 2.13 (0.36, 12.57) | 0.400 | 1.45 (0.86, 2.45) | 0.16 | 33% |
| The analysis is adjusted for age, sex, and region. Percentage mediated only calculated when direct and indirect effect estimates are in the same direction. HHV-6, human herpesvirus 6. Results in boldface denote statistical significance (p<0.05). The A2‐module included 687 CpGs across 627 genes. | | | | | | | | | |

## **Table S8.** Association between HHV-6 DNA positivity, anti-CMV IgG and estimated blood cell-type composition.

| **Exposure** | **Outcome** | **AOR (95%CI)** | **P-value** |
| --- | --- | --- | --- |
| HHV-6 DNA positivity | CD8T | -0.008 (-0.022, 0.006) | 0.27 |
| HHV-6 DNA positivity | CD4T | **0.017 (0.001, 0.033)** | **0.036** |
| HHV-6 DNA positivity | NK | -0.005 (-0.012, 0.003) | 0.224 |
| HHV-6 DNA positivity | B cell | 0.004 (-0.001, 0.010) | 0.134 |
| HHV-6 DNA positivity | Monocyte | -0.006 (-0.014, 0.001) | 0.074 |
| HHV-6 DNA positivity | Granulocyte | -0.002 (-0.028, 0.023) | 0.852 |
| CMV IgG (≥1 vs <1) | CD8T | **0.010 (0.002, 0.018)** | **0.019** |
| CMV IgG (≥1 vs <1) | CD4T | **-0.014 (-0.024, -0.005)** | **0.003** |
| CMV IgG (≥1 vs <1) | NK | **0.006 (0.002, 0.011)** | **0.004** |
| CMV IgG (≥1 vs <1) | B cell | **-0.004 (-0.007, -0.000)** | **0.029** |
| CMV IgG (≥1 vs <1) | Monocyte | **-0.005 (-0.010, -0.001)** | **0.013** |
| CMV IgG (≥1 vs <1) | Granulocyte | 0.007 (-0.008, 0.022) | 0.358 |
| The analysis is adjusted for age, sex, and region. HHV-6, human herpesvirus 6; CD8T, CD8⁺ T cells; CD4T, CD4⁺ T cells; NK, natural killer cells. Results in boldface denote statistical significance (p<0.05). | | | |

## **Table S9.** Associations between viral indices and multiple sclerosis risk: evaluation of DNAm-modules as mediators across immune cell types.

| **Exposure** | **Mediator** | **Outcome** | **Total effect** | | **Direct effect** | | **Indirect effect** | | % mediated |
| --- | --- | --- | --- | --- | --- | --- | --- | --- | --- |
|  |  |  | AOR (95%CI) | P-value | AOR (95%CI) | P-value | AOR (95%CI) | P-value |  |
| HHV-6 DNA (positive vs negative) | A2-module - CD8T | MS onset | **3.05 (1.26, 7.41)** | **0.014** | 2.03 (0.85, 4.85) | 0.110 | **1.50 (1.08, 2.08)** | **0.014** | 37% |
| HHV-6 DNA (positive vs negative) | A2-module - CD4T | MS onset | **3.21 (1.2, 8.56)** | **0.020** | 2.1 (0.78, 5.61) | 0.140 | **1.53 (1.02, 2.29)** | **0.039** | 37% |
| HHV-6 DNA (positive vs negative) | A2-module - NK | MS onset | **3.08 (1.24, 7.66)** | **0.015** | 2.86 (1.19, 6.9) | 0.019 | 1.08 (0.91, 1.27) | 0.380 | 6% |
| HHV-6 DNA (positive vs negative) | A2-module - B cell | MS onset | **3.26 (1.22, 8.74)** | **0.019** | 2.67 (0.96, 7.45) | 0.061 | 1.22 (0.88, 1.69) | 0.220 | 17% |
| HHV-6 DNA (positive vs negative) | A2-module - monocyte | MS onset | **3.1 (1.26, 7.65)** | **0.014** | 2.03 (0.87, 4.7) | 0.100 | **1.53 (1.07, 2.19)** | **0.019** | 38% |
| HHV-6 DNA (positive vs negative) | A2-module - granulocyte | MS onset | **3.11 (1.25, 7.74)** | **0.015** | 1.97 (0.81, 4.76) | 0.13 | **1.58 (1.1, 2.26)** | **0.013** | 40% |
| CMV IgG (≥1 vs <1) | A4-module - CD8T | MS onset | 0.75 (0.48, 1.18) | 0.22 | **0.62 (0.4, 0.96)** | **0.033** | **1.21 (1.05, 1.4)** | **0.0091** | - |
| CMV IgG (≥1 vs <1) | A4-module - CD4T | MS onset | 0.75 (0.48, 1.18) | 0.22 | **0.58 (0.38, 0.9)** | **0.016** | **1.28 (1.05, 1.57)** | **0.016** | - |
| CMV IgG (≥1 vs <1) | A4-module - NK | MS onset | 0.75 (0.48, 1.18) | 0.21 | 0.73 (0.47, 1.14) | 0.17 | 1.03 (0.94, 1.12) | 0.51 | - |
| CMV IgG (≥1 vs <1) | A4-module - B cell | MS onset | 0.75 (0.48, 1.18) | 0.21 | 0.66 (0.41, 1.04) | 0.072 | **1.14 (1.01, 1.29)** | **0.032** | - |
| CMV IgG (≥1 vs <1) | A4-module - monocyte | MS onset | 0.75 (0.48, 1.18) | 0.22 | **0.6 (0.39, 0.93)** | **0.022** | **1.25 (1.06, 1.48)** | **0.0074** | - |
| CMV IgG (≥1 vs <1) | A4-module - granulocyte | MS onset | 0.75 (0.48, 1.18) | 0.22 | **0.58 (0.38, 0.9)** | **0.016** | **1.29 (1.06, 1.56)** | **0.011** | - |
| The analysis is adjusted for age, sex, and region. Percentage mediated only calculated when direct and indirect effect estimates are in the same direction. HHV-6, human herpesvirus 6; CD8T, CD8⁺ T cells; CD4T, CD4⁺ T cells; NK, natural killer cells. Cell-type–specific methylation mediators were derived using Tensor Composition Analysis (TCA), with the first principal component (PC1) of DNA methylation within the A2-module CpG set used as the summary score for each cell type. Results in boldface denote statistical significance (p<0.05). The A2‐module included 687 CpGs across 627 genes | | | | | | | | | |

## **Table S10.** The associations between human herpesvirus indices and multiple sclerosis with further consideration of the A2-module as a mediating factor, with mediation effects modified by HLA allele status.

| **Exposure** | **Mediator** | **Outcome** | **Estimate** | **Non-risk** | | | **Risk** | | | **Interaction** | |
| --- | --- | --- | --- | --- | --- | --- | --- | --- | --- | --- | --- |
|  |  |  |  | AOR (95%CI) | P-value | % mediated | AOR (95%CI) | P-value | % mediated | AOR (95%CI) | P-value |
| **Combined HLA susceptibility** | | | | | | | | | | | |
| HHV-6 DNA (positive vs negative) | A2-module | MS onset | Indirect | **2.06 (1.38, 3.07)** | **0.001** | 63% | 1.05 (0.62, 1.77) | 0.857 | 10% | **0.51 (0.27, 0.97)** | **0.041** |
| HHV-6 DNA (positive vs negative) | A2-module | MS onset | Direct | 1.53 (0.37, 6.39) | 0.558 |  | 1.53 (0.37, 6.39) | 0.558 |  |  |  |
| HHV-6 DNA (positive vs negative) | A2-module | MS onset | Total | 3.15 (0.66, 14.96) | 0.148 |  | 1.61 (0.34, 7.61) | 0.549 |  |  |  |
| ***HLA-A:02*** | | | | | | | | | | | |
| HHV-6 DNA (positive vs negative) | A2-module | MS onset | Indirect | 2.02 (0.97, 4.20) | 0.060 | 74% | 1.52 (0.96, 2.40) | 0.075 | 0.63% | 0.75 (0.32, 1.79) | 0.519 |
| HHV-6 DNA (positive vs negative) | A2-module | MS onset | Direct | 1.28 (0.19, 8.60) | 0.801 |  | 1.28 (0.19, 8.60) | 0.801 |  |  |  |
| HHV-6 DNA (positive vs negative) | A2-module | MS onset | Total | 2.58 (0.24, 27.53) | 0.433 |  | 1.94 (0.29, 13.03) | 0.496 |  |  |  |
| ***HLA-DRB1*1501*** | | | | | | | | | | | |
| HHV-6 DNA (positive vs negative) | A2-module | MS onset | Indirect | **2.05 (1.31, 3.20)** | **0.002** | 52% | 1.25 (0.74, 2.12) | 0.401 | 26% | 0.61 (0.31, 1.22) | 0.160 |
| HHV-6 DNA (positive vs negative) | A2-module | MS onset | Direct | 1.92 (0.33, 11.31) | 0.472 |  | 1.92 (0.33, 11.31) | 0.472 |  |  |  |
| HHV-6 DNA (positive vs negative) | A2-module | MS onset | Total | 3.93 (0.58, 26.40) | 0.160 |  | 2.40 (0.40, 14.30) | 0.335 |  |  |  |
| The analysis is adjusted for age, sex, and region. In all models, the full region variable with four levels was used, except for the model involving HHV-6 DNA (positive vs negative), A2-module, MS onset and HLAA2bin as the moderator where categories 2 and 3 were collapsed due to sparse cells. *HLA-DRB1*1501*=rs3135388: AA/AG, susceptible vs GG, non-susceptible; *HLA-A:02*=rs2844821: AA, susceptible vs AG, non-susceptible; and combined HLA susceptibility=individuals with both *HLA-DRB1*1501* AA/AG and *HLA-A:02* AA genotypes, susceptible vs all others, non-susceptible. HHV-6, human herpesvirus 6. Results in boldface denote statistical significance (p<0.05). The A2‐module included 687 CpGs across 627 genes. | | | | | | | | | | | |

## **Supplementary Analysis**

*Excluding participants with high HHV-6 DNA loads.*

Two participants, both cases, had HHV-6 DNA loads>1.0x10^6^ a threshold used to identify inherited chromosomally integrated HHV-6. A sensitivity analysis was conducted where these two cases were excluded from the analysis. This did not alter the results (data not shown).

*The association between anti-CMV IgG and multiple sclerosis with further consideration of A4-module as a mediating factor.*

Formal mediation analysis was conducted to evaluate the extent to which HHV infection or host response could be operating through the DNAm-modules to alter MS risk. Higher CMV IgG titer levels were positively associated with A4-module, which in turn was associated with increased MS onset risk. However, the direct (OR=0.62 (95%CI=0.40-0.96, p=0.033)) and indirect (OR=1.22 (95%CI=1.04-1.43, p=0.013)) effects were in opposite directions (**Table S4**).

The CMV mediation result persisted after restricting to FDE cases (OR_indirect effect_ = 1.23 (95%CI: 1.02-1.48 ), p = 0.032) and excluding DMT treated cases (OR_indirect effect_ = 1.82 (95%CI: 1.16-2.85), p = 0.020). Reverse causation testing demonstrated the CMV IgG findings to be less clear, again partly because the direct and indirect effects were in opposite directions (OR_direct effect_ = 0.61(95%CI: 0.38-0.98), p = 0.039; OR_indirect effect_ = 1.24 (95%CI: 1.06-1.46), p = 0.008). We tested if the A4-module gene set was significantly enriched for GWAS MS risk genes. For the 29 genes in the A4-module, we observed increasing overlap at higher-ranked genes. Specifically, the strongest enrichment was observed among the top-ranked genes (lowest p = 0.0499; fold enrichment = 19.8), while the signal decreased as larger fractions of the module were included, indicating that epigenetic changes may precede clinical onset, as the top module genes are genetically linked to MS. Among the A4-module CpGs set, 34.4% had at least one previously reported significant cis-meQTL in the EPIC meQTL resource, a percentage that is similar to the previously published EPIC methylome that is under genetic control^24^. These CpGs therefore have evidence of nearby genetic variants associated with variation in DNA methylation levels in peripheral blood.

Higher CMV IgG titer levels were associated with all estimated cell types except granulocytes (**Table S8**). Cell type specific methylation PC1 scores derived using TCA showed that mediation of the association between HHV-6 DNA positivity and the MS risk was observed in all cell specific compartments except for the NK cell compartment (**Table S9**).

## **Supplementary Box 1. Investigation approaches to assess the role of the A4-module in CMV infection and MS risk.**

There appeared to be statistical suppression^1^ of the A4-module in the association between higher CMV IgG and MS onset. This is a situation where direct and indirect effects point in opposite directions^2,3^. It is not unusual to have environmental exposure working through multiple pathways with different effects^4^. In our study the natural direct effect reflects the portion of CMV's effect on MS risk that occurs independently of DNAm, where DNAm is held constant at the level it would naturally take in the absence of CMV infection. The reduced risk of MS onset suggested by the direct effect supports the findings of past observational studies^5,6^ that CMV infection may be protective in MS. Further, mechanistic evidence has indicated that CMV shapes NKG2C^+^ NK cell compartments that better control EBV-driven autoreactive cells^7^, providing a plausible mechanism by which CMV lowers MS risk without involving DNAm. Future studies may address the direct effect and to explore additional mediators, prioritising those likely to yield a protective indirect pathway.

On the other hand, the indirect effect, which represents the pathway through which CMV affects MS risk via DNAm, indicated an increased risk of MS. In human cohorts, CMV infection has been linked to widespread alterations in DNAm patterns^8^ and to accelerated epigenetic aging^9,10^. These epigenetic features represent recognized risk factors for MS^11,12^. Although an adverse effect of CMV infection on MS risk through DNAm has not previously been demonstrated, CMV infection has been suggested to promote cardiovascular disease through epigenetic programming^14^. The A4-module enrichment analyses demonstrated relevance to MS (**Figure S1**). IKZF1 was the top rank TF for the A4-module and is known to have a role in lymphoid development^15^, cells that have been implicated in MS pathogenesis^16,17^. PARP-1, the 3^rd^ ranked upstream transcription factor of the A4-module, is activated by CMV and essential for its replication^18,19^. PARP-1 is also implicated in the pathogenesis of MS^20-22^, with PARP-1 inhibitors proposed as a potential MS treatment^23^. These features highlight the immune related nature of the A4-module. However, for CMV, biological pathways independent of the A4-module also appear important.

These results indicate that CMV showed complex associations with MS onset risk involving the A4-module.

*Examining whether the indirect effect of anti-CMV IgG on MS onset operating through the A4-module differ by HLA genotype.*

We assessed whether the observed DNAm mediation effects of anti-CMV IgG differed by *HLA-DRB1*1501, HLA-A:02* and variants. The indirect effect for the association between higher anti-CMV IgG and MS onset, operating through the A4-module, did not differ by any of the two HLA genotype groups (*HLA-DRB1*1501* OR_interaction_= 0.95 (95%CI:0.68-1.32), p= 0.053; *HLA-A:02* OR_interaction_= 0.84 (95%CI: 0.59-1.20), p = 0.064) or those with both the *HLA-DRB1*1501* and *HLA-A:02* risk genotypes compared to those with all other combinations (OR_interaction_= 0.87 (95%CI: 0.59-1.28), p = 0.030).

*Biological relevance of A4-module.*

The A4-module comprises 29 CpGs across 29 genes. The enrichment analysis for this module’s gene set in the Reactome database produced only results where a singular gene was included in the pathway. When performing the same analysis with the Gene Ontology database as the reference, only one enriched pathway was up/down regulated by 2 or more genes in the module set: *Protein tyrosine kinase binding* (fold enrichment 64.9). Using WebCSEA we sought to analyse the adult tissue and cell types potentially affected by disruptions in the A4-module gene set. The A4-module was not enriched for any organ system cell-types marker genes, indicating the A4-module had no apparent association with organ system cell-type marker genes. To explore potential regulatory mechanisms linking the A4-module to MS, we identified the top ten transcription factors associated with the A4-module. Of the top 10 transcription factors linked to the A4-module (**Figure S2**), four were associated with MS. These included IKZF1, PARP1, MED1 and HNF1A.


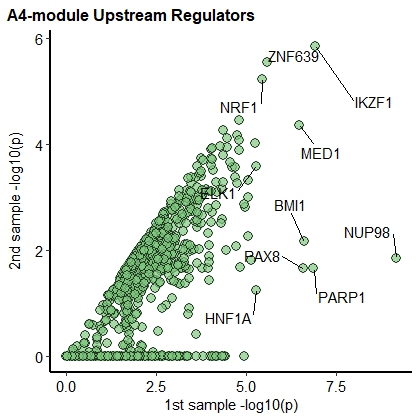


## **Figure S1.** Upstream regulators of A4-module gene set inferred using the LISA platform. The A4-module included 29 CpGs across 29 genes. Because ranking is performed independently for each transcription factor, the 1st and 2nd samples may correspond to different ChIP-seq experiments across transcription factors.

*Replication of A4-module*

To validate our findings, we investigated whether the associations between modules identified as mediators and MS case status would replicate in another MS case-control study (29 MS patients and 18 controls recruited from clinical practice at the University of Illinois, Chicago) with comparable DNAm measures (whole blood Illumina 850k DNAm)^25^. The modules were reconstructed (represented using its first PC) and associations with MS case status were tested using logistic regression, adjusting for age, sex, the first two PCs from the full methylation dataset and estimated cell type proportions (B cells, NK cells, CD4^+^ T cells, CD8^+^ T cells, monocytes, neutrophils, eosinophils) using the EpiDISH R package. Empirical p-values were derived by repeating the analysis with 1000 random CpG sets (matched to DNAm-module size, sampled from the 850k array), extracting PC1 from each, and comparing their associations with MS, the significance defined by the proportion of analyses with measures of association as or stronger than those using our DNAm module CpGs. This analysis was undertaken for A4-module, as we previously demonstrated that the biological relevance of the A2-module showed substantial overlap with modules in the Epidemiological Investigation of Multiple Sclerosis (EIMS) study^13^.

In this independent whole blood dataset the A4-module (represented using PC1) was strongly associated with MS case status (OR=286.56 (95%CI=1.55-53052.59, p=0.034)) and of 1000 randomised A4-modules, only 3% of p-values were as or more extreme than those observed (**Figure S2**). Importantly, for the first time, we found evidence of external replication for the A4-module in support of the A4-module as a replicated epigenetic marker of MS risk.


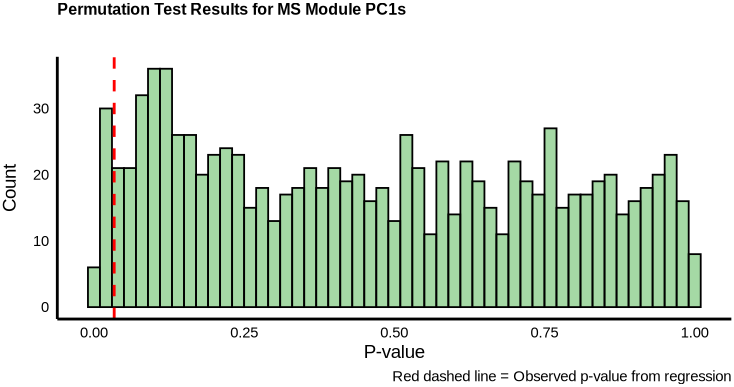


**Figure S2.** Distribution of p-values for the association between randomised A4-module and MS onset risk. Analysis undertaken in an independent whole blood DNA methylation dataset (29 MS patients and 18 controls).

**Supplemental references**

1. MacKinnon DP, Krull JL, Lockwood CM. Equivalence of the mediation, confounding and suppression effect. *Prev Sci*. Dec 2000;1(4):173-81. doi:10.1023/a:1026595011371

2. Hair JF, Hult GTM, Ringle CM, Sarstedt M, Danks NP, Ray S. Mediation Analysis. In: Hair Jr JF, Hult GTM, Ringle CM, Sarstedt M, Danks NP, Ray S, eds. *Partial Least Squares Structural Equation Modeling (PLS-SEM) Using R: A Workbook*. Springer International Publishing; 2021:139-153.

3. Zhao X, Lynch Jr JG, Chen Q. Reconsidering Baron and Kenny: Myths and truths about mediation analysis. *Journal of consumer research*. 2010;37(2):197-206.

4. Wu H, Eckhardt CM, Baccarelli AA. Molecular mechanisms of environmental exposures and human disease. *Nat Rev Genet*. May 2023;24(5):332-344. doi:10.1038/s41576-022-00569-3

5. Grut V, Biström M, Salzer J, et al. Cytomegalovirus seropositivity is associated with reduced risk of multiple sclerosis-a presymptomatic case-control study. *Eur J Neurol*. Sep 2021;28(9):3072-3079. doi:10.1111/ene.14961

6. Sundqvist E, Bergström T, Daialhosein H, et al. Cytomegalovirus seropositivity is negatively associated with multiple sclerosis. *Mult Scler*. Feb 2014;20(2):165-73. doi:10.1177/1352458513494489

7. Vietzen H, Berger SM, Kühner LM, et al. Ineffective control of Epstein-Barr-virus-induced autoimmunity increases the risk for multiple sclerosis. *Cell*. Dec 21 2023;186(26):5705-5718.e13. doi:10.1016/j.cell.2023.11.015

8. Bergstedt J, Azzou SAK, Tsuo K, et al. The immune factors driving DNA methylation variation in human blood. *Nat Commun*. Oct 6 2022;13(1):5895. doi:10.1038/s41467-022-33511-6

9. Poloni C, Szyf M, Cheishvili D, Tsoukas CM. Are the Healthy Vulnerable? Cytomegalovirus Seropositivity in Healthy Adults Is Associated With Accelerated Epigenetic Age and Immune Dysregulation. *J Infect Dis*. Feb 1 2022;225(3):443-452. doi:10.1093/infdis/jiab365

10. Kananen L, Nevalainen T, Jylhävä J, et al. Cytomegalovirus infection accelerates epigenetic aging. *Exp Gerontol*. Dec 2015;72:227-9. doi:10.1016/j.exger.2015.10.008

11. Liu H, Zhang H, Yin Z, Hou M. Assessment of relationships between epigenetic age acceleration and multiple sclerosis: a bidirectional mendelian randomization study. *Epigenetics Chromatin*. Jan 30 2025;18(1):7. doi:10.1186/s13072-025-00567-9

12. Xavier A, Maltby VE, Ewing E, et al. DNA Methylation Signatures of Multiple Sclerosis Occur Independently of Known Genetic Risk and Are Primarily Attributed to B Cells and Monocytes. *Int J Mol Sci*. Aug 8 2023;24(16)doi:10.3390/ijms241612576

13. Simpson-Yap S, Morwitch E, Tanner SA, et al. Epstein–Barr Virus, Lower Vitamin D, Low Sun Exposure, and HLA-DRB1*1501 Risk Variant Share Common Epigenetic Pathways Leading to Multiple Sclerosis Onset. *Annals of Neurology*. n/a(n/a)doi:<https://doi.org/10.1002/ana.78043>

14. Giacconi R, Pirazzini C, Bacalini MG, et al. Association of cytomegalovirus serostatus with ELOVL2 methylation: Implications for lipid metabolism, inflammation, DNA damage, and repair capacity in the MARK-AGE study population. *Mech Ageing Dev*. Jun 2025;225:112043. doi:10.1016/j.mad.2025.112043

15. Rahmani M, Fardi M, Farshdousti Hagh M, Hosseinpour Feizi AA, Talebi M, Solali S. An investigation of methylation pattern changes in the IKZF1 promoter in patients with childhood B-cell acute lymphoblastic leukemia. *Blood Res*. Jun 2019;54(2):144-148. doi:10.5045/br.2019.54.2.144

16. van Langelaar J, Rijvers L, Smolders J, van Luijn MM. B and T Cells Driving Multiple Sclerosis: Identity, Mechanisms and Potential Triggers. *Front Immunol*. 2020;11:760. doi:10.3389/fimmu.2020.00760

17. Sadeghi Hassanabadi N, Broux B, Marinović S, Gotthardt D. Innate Lymphoid Cells - Neglected Players in Multiple Sclerosis. *Front Immunol*. 2022;13:909275. doi:10.3389/fimmu.2022.909275

18. Chung WC, Song MJ. Virus-Host Interplay Between Poly (ADP-Ribose) Polymerase 1 and Oncogenic Gammaherpesviruses. *Front Microbiol*. 2021;12:811671. doi:10.3389/fmicb.2021.811671

19. Zhang W, Guo J, Chen Q. Role of PARP-1 in Human Cytomegalovirus Infection and Functional Partners Encoded by This Virus. *Viruses*. Sep 15 2022;14(9)doi:10.3390/v14092049

20. Farez MF, Quintana FJ, Gandhi R, Izquierdo G, Lucas M, Weiner HL. Toll-like receptor 2 and poly(ADP-ribose) polymerase 1 promote central nervous system neuroinflammation in progressive EAE. *Nat Immunol*. Sep 2009;10(9):958-64. doi:10.1038/ni.1775

21. Chiarugi A. Inhibitors of poly(ADP-ribose) polymerase-1 suppress transcriptional activation in lymphocytes and ameliorate autoimmune encephalomyelitis in rats. *Br J Pharmacol*. Nov 2002;137(6):761-70. doi:10.1038/sj.bjp.0704934

22. Scott GS, Kean RB, Mikheeva T, et al. The therapeutic effects of PJ34 [N-(6-oxo-5,6-dihydrophenanthridin-2-yl)-N,N-dimethylacetamide.HCl], a selective inhibitor of poly(ADP-ribose) polymerase, in experimental allergic encephalomyelitis are associated with immunomodulation. *J Pharmacol Exp Ther*. Sep 2004;310(3):1053-61. doi:10.1124/jpet.103.063214

23. Meira M, Sievers C, Hoffmann F, et al. PARP-1 deregulation in multiple sclerosis. *Mult Scler J Exp Transl Clin*. Oct-Dec 2019;5(4):2055217319894604. doi:10.1177/2055217319894604

24. Villicaña S, Bell JT. Genetic impacts on DNA methylation: research findings and future perspectives. *Genome Biol*. Apr 30 2021;22(1):127. doi:10.1186/s13059-021-02347-6

25. Bingen JM, Clark LV, Band MR, Munzir I, Carrithers MD. Differential DNA methylation associated with multiple sclerosis and disease modifying treatments in an underrepresented minority population. *Front Genet*. 2022;13:1058817. doi:10.3389/fgene.2022.1058817
